# Contemporary Theory and Research

**Published:** 1893-08-26

**Authors:** 


### Contemporary Theory and Research.

### DEFECTIVE EYESIGHT IN LAUSANNE.

Dr. Eperon has published some interesting statistics relating to the eyesight of the pupils attending the various schools at Lausanne. The results are rather startling, and seem to point to some grave defect in the hygienic conditions under which the children pursue their education. In the primary schools, where the ages vary from 8 to 14, the cases of defective vision are found to be 27 per cent. both for boys and girls. In the higher schools the proportion is about 22 or 23 per cent., but it is remarkable that the proportion of those afflicted with myopia rises steadily with the degree of instruction provided at the school. Among young children more girls than boys are short-sighted, but this inequality is reversed in the higher schools. Dr. Eperon points out that the population of the schools falls into two great groups—those of Romand and those of German origin; it is principally among the latter that he finds the cases of myopia, due he considers to the hereditary effect of the use of Gothic characters. He considers much might be done by the use of good school apparatus and the introduction of upright writing, to reduce the percentage of those affected.

### ICED WATER.

The *Lancet* has subjected six specimens of ice from London depôts to chemical and bacteriological analysis with the following result: "(1) By far the greater proportion of ice supplied in London is natural (generally Norwegian). Of the specimens procured only one had been produced artificially, and this specimen gave indifferent results on chemical analysis, but results of an eminently satisfactory kind in the light of bacteriological inquiry, practically no development of colonies of organisms taking place on culture. (2) Two out of five specimens of ice imported into this country from Norway, whilst yielding a satisfactory chemical analysis, were decidedly bad according to bacteriological examination, the number of colonies of organisms counted on culture varying from 400 to 700 per cubic centimetre of the melted ice. (3) Three out of five specimens of imported ice, though furnishing no condemnatory evidence on chemical examination yielded bacteriological results such as might under certain circumstances give rise to suspicion, though they may be regarded as of fairly good quality." The conclusion is, of course, that ice used for drinking purposes should be produced by the artificial freezing of distilled or sterilized water, but this is more easily said than done, in the average household at any rate, where the water drinker is still at a cruel disadvantage compared with the rest of the community.

### AMOK.

This is a remarkable form of homicidal mania, to which, according to Dr. Gilmore Ellis, the Malays are subject. When under the influence of this passion, which appears to seize them without warning, the Malay "runs armed through the most crowded street or village stabbing right and left at man, woman, or child, relation, friend, or stranger." After the period of excitement is over, the patient or murderer, which ever it is decided to call him, loses all memory of what has occurred, and subsides into a sullen and apathetic condition. A wild stare in the eyes is a usual symptom, and on being questioned they many of them speak of having seen everything red shortly before the attack, when they lost the consciousness of what they were doing. Dr. Ellis refers the phenomena to masked epilepsy, strong emotion bringing on a sudden paroxysm of acute homicidal mania, due to disturbances in the sensory centres. It is curious that from this disease we get the expression, "to run amuck," or, as it should be, "to run amok."

### INNOCUOUS MICRO-ORGANISMS.

Dr. Liston, in a paper read before the Incorporated Society of Medical Officers of Health, raises the theory that "it only requires certain micro-organisms from outside sources to be brought into contact with those in the human system to cause fever and other germs to spring into activity and develop these several diseases we call 'specific fevers.' Professor Brown had stated that he found thirteen different micro-organisms in pure milk from healthy cows, and collected with all antiseptic precautions. If he found this number of micro-organisms in healthy milk and collected as he stated, there is nothing more likely than that our food contains numerous micro-organisms that we are not even acquainted with. From experiments made on dogs and other animals, it has been stated that diseases may be produced in them by inoculation with the infectious disease occurring in man, and it has been proved that unless inoculated with innocuous micro-organisms they do not prove fatal. The most interesting experiments on the subject were made by Messrs. Vaillard and Vincent, who found that when a culture of tetanus so attenuated as not to produce tetanus is inoculated with the microbe bacillus prodigiosus, the animal invariably dies with well-marked tetanic spasms. It has also been experimentally proved that chemical poisons secreted by a certain microbe, if injected into an animal, predispose it to infection by the same micro-organism. Need we wonder, then, that the presence in man of the tubercle of typhoid bacillus, or the pneumococcus, predisposes to the infection by the ordinary microbes of putrefaction and the generalization of specific microbes causing the disease?"

